# Draft Genome Sequence of a High-Level Colistin-Resistant Clinical Strain of the *Enterobacter cloacae* Complex

**DOI:** 10.1128/genomeA.00131-17

**Published:** 2017-04-06

**Authors:** Ji Lin, Feifei Zhao, Yu Feng, Zhiyong Zong

**Affiliations:** a Center of Infectious Diseases, West China Hospital, Sichuan University, Chengdu, China; b Division of Infectious Diseases, State Key Laboratory of Biotherapy, Chengdu, China; c Department of Infection Control, West China Hospital, Sichuan University, Chengdu, China

## Abstract

Strain WCHECl-C4 of the* Enterobacter cloacae* complex, recovered from the blood of a patient with peritonitis, was high-level resistant to colistin. Here, we report its 5.1-Mb draft genome sequence, comprising 92 contigs with an average 55.74% G+C content. The genome contained 4,783 coding sequences and 68 tRNA genes.

## GENOME ANNOUNCEMENT

The *Enterobacter cloacae* complex comprises bacterial species such as *E. asburiae*, *E. cancerogenus*, and *E. cloacae*, which are common pathogens of human infections (1). Strain WCHECl-C4 was recovered from the blood of a 52-year-old female patient with peritonitis at West China Hospital, Chengdu, China, in February 2015. Strain WCHECl-C4 was identified as *E. cloacae* by Vitek II (bioMérieux, Lyon, France) and is highly resistant to colistin (MIC, >128 μg/mL) but is susceptible to imipenem (MIC, 0.125 μg/mL), as determined using the microdilution broth method (2). Strain WCHECl-C4 did not have the plasmid-borne colistin resistance genes *mcr-1* and *mcr-2*, which were screened by PCR (3, 4).

Whole-genome sequencing of strain WCHECl-C4 was performed using the HiSeq 2500 Sequencer (Illumina, San Diego, CA, USA) with the 150-bp paired-end protocol and 200× coverage, which generated 1.28 Gb of clean bases and 4,265,547 reads. Using SPAdes (5), 92 contigs (69 contigs ≥ 1,000 bp in length; *N*_50_, 157,468 bp) with a 55.74% G+C content were assembled. Prokka (6) was used to annotate the genome, which was about 5.1 Mb and had 4,783 coding sequences and 68 tRNA genes. Strain WCHECl-C4 belonged to ST414 (*dnaA*, *fusA*, *gyrB*, *leuS*, *pyrG*, *rplB*, and *rpoB*; 136-99-153-160-140-12-92), which was determined using the multilocus sequence typing tool of the Center for Genomic Epidemiology (http://genomicepidemiology.org).

Antimicrobial resistance genes were predicted using ResFinder from the Center for Genomic Epidemiology, and only three antimicrobial resistance genes, *bla*_ACT_ (encoding an AmpC β-lactamase conferring resistance to cephalosporins), *qnrS1* (conferring low-level resistance to quinolones), and *fosA* (conferring fosfomycin resistance) were identified in strain WCHECl-C4. The *bla*_ACT_ gene of strain WCHECl-C4 is chromosomally located and encodes an ACT enzyme that has not been assigned a number yet. The mechanism of colistin resistance in strain WCHECl-C4 remains unclear and warrants further investigation. PlasmidFinder from the Center for Genomic Epidemiology predicted that strain WCHECl-C4 had no plasmid replicons known to the *Enterobacteriaceae*.

To further determine the species of WCHECl-C4, the JSpecies web program (http://imedea.uib-csic.es/jspecies) with default settings (7) was used to determine the pairwise average nucleotide identities (ANIs) between strain WCHECl-C4 and the type or reference (if the genome of type strain was not available) strain of each species of the *E. cloacae* complex. The type or reference strains used were *E. asburiae* JCM 6051^T^ (GenBank accession no. BBED00000000), *E. mori* LMG 25706^T^, (AEXB00000000), *E. cloacae* subsp. *cloacae* ATCC 13047, (CP0019180), *E. ludwigii* EN-119^T^ (CP017279), *E. hormaechei* ATCC 49162 (AFHR00000000), *E. xiangfangensis* LMG27195^T^ (CP017183), *E. cancerogenus* ATCC 35316 (ABWM00000000), and *E. soli* ATCC BAA-2102^T^ (LXES00000000). The genome of strain WCHECl-C4 shared only 82.25% to 92.56% ANIs with the type and reference strains of the *E. cloacae* complex, which were below the ≥95% ANI cutoff to define a bacterial species (8), suggesting that strain WCHECl-C4 is likely to belong to a new species of the *E. cloacae* complex.

### Accession number(s).

This whole-genome shotgun project has been deposited at DDBJ/EMBL/GenBank under the accession number MTSO00000000. The version described in this paper is the first version, MTSO01000000.

## References

[1] Mezzatesta ML, Gona F, Stefani S 2012 *Enterobacter cloacae* complex: clinical impact and emerging antibiotic resistance. Future Microbiol 7:887–902. doi:10.2217/fmb.12.61. 22827309

[2] Clinical and Laboratory Standards Institute 2013 Performance standards for antimicrobial susceptibility testing. CLSI publication M100–S23. Clinical and Laboratory Standards Institute, Wayne, PA.

[3] Xavier BB, Lammens C, Ruhal R, Kumar-Singh S, Butaye P, Goossens H, Malhotra-Kumar S 2016 Identification of a novel plasmid-mediated colistin-resistance gene, *mcr-2*, in *Escherichia coli*, Belgium, June 2016. Euro Surveill 21:30280. doi:10.2807/1560-7917.ES.2016.21.27.30280. 27416987

[4] Liu YY, Wang Y, Walsh TR, Yi LX, Zhang R, Spencer J, Doi Y, Tian G, Dong B, Huang X, Yu LF, Gu D, Ren H, Chen X, Lv L, He D, Zhou H, Liang Z, Liu JH, Shen J 2016 Emergence of plasmid-mediated colistin resistance mechanism MCR-1 in animals and human beings in China: a microbiological and molecular biological study. Lancet Infect Dis 16:161–168. doi:10.1016/S1473-3099(15)00424-7. 26603172

[5] Bankevich A, Nurk S, Antipov D, Gurevich AA, Dvorkin M, Kulikov AS, Lesin VM, Nikolenko SI, Pham S, Prjibelski AD, Pyshkin AV, Sirotkin AV, Vyahhi N, Tesler G, Alekseyev MA, Pevzner PA 2012 SPAdes: a new genome assembly algorithm and its applications to single-cell sequencing. J Comput Biol 19:455–477. doi:10.1089/cmb.2012.0021. 22506599 PMC3342519

[6] Seemann T 2014 Prokka: rapid prokaryotic genome annotation. Bioinformatics 30:2068–2069. doi:10.1093/bioinformatics/btu153. 24642063

[7] Richter M, Rosselló-Móra R 2009 Shifting the genomic gold standard for the prokaryotic species definition. Proc Natl Acad Sci U S A 106:19126–19131. doi:10.1073/pnas.0906412106. 19855009 PMC2776425

[8] Goris J, Konstantinidis KT, Klappenbach JA, Coenye T, Vandamme P, Tiedje JM 2007 DNA-DNA hybridization values and their relationship to whole-genome sequence similarities. Int J Syst Evol Microbiol 57:81–91. doi:10.1099/ijs.0.64483-0. 17220447

